# Investigation of the association between the antibody responses to neurotropic viruses and dementia outcomes in the UK Biobank

**DOI:** 10.1371/journal.pone.0274872

**Published:** 2022-10-12

**Authors:** Krisztina Mekli, Artitaya Lophatananon, Rachel Cant, Alistair Burns, Curtis B. Dobson, Ruth F. Itzhaki, Kenneth R. Muir

**Affiliations:** 1 Division of Population Health, Health Services Research and Primary Care, School of Health Sciences, Faculty of Biology, Medicine and Health, The University of Manchester, Manchester, United Kingdom; 2 Division of Pharmacy & Optometry, School of Health Sciences, Faculty of Biology, Medicine and Health, The University of Manchester, Manchester, United Kingdom; 3 Division of Neuroscience & Experimental Psychology, School of Biological Sciences, Faculty of Biology, Medicine and Health, The University of Manchester, Manchester, United Kingdom; 4 The Oxford Institute of Population Ageing, University of Oxford, Oxford, United Kingdom; University Medical Center Rotterdam, NETHERLANDS

## Abstract

The causes that trigger the onset of dementia are still unknown. Recently there has been an increasing interest in the possible role of infectious agents in the brain in the pathogenesis of this condition. Amongst the viruses, members of the *Herpesviridae* family, namely herpes simplex virus-1 (HSV1), cytomegalovirus (CMV), human herpesvirus-6 (HHV6), human herpesvirus-7 (HHV7) and varicella zoster virus (VZV) have been suggested as potential causes of the disease. However, the relative importance of these and other viruses in contributing to dementia remains unclear. We evaluated the association between seropositivity status of all viruses available in a large, population-based dataset (the UK Biobank) and dementia risk in an unbiased way. Of the 15 viruses investigated, our results showed a statistically significant increase of dementia risk associated only with HSV1 seropositivity (OR 2.14, 95% C.I. 1.21–3.81). However, by combining the data we found that seropositivity for 4 viruses (HSV1, HHV6, HHV7 and VZV) also significantly increases the risk of dementia (OR = 2.37, 95% C.I. 1.43–3.92). These four viruses have been described previously as neurotropic viruses. Our results provide support for a role for neurotropic viruses in the pathology of dementia.

## Introduction

As the population ages and people live longer, dementia has become one of the most important health challenges in the world. The World Health Organisation estimates that around 50 million people have dementia worldwide and every year there are nearly 10 million new cases. The estimated proportion of the general population aged 60 and over with dementia at a given time is between 5–8%. The total number of people with dementia is projected to reach 82 million in 2030 and 152 in 2050 [1].

In the UK, there are currently about 850,000 people living with dementia, and the number is projected to reach 1.6 million by 2040, according to the Alzheimer’s Society [2]. The total cost of care for people with dementia is £34.7 billion and is set to rise over the next two decades to £94.1 billion. Apart from the economic impact, there is a societal one as well. The NHS estimates that one in three people will care for a person with dementia in their lifetime [3].

Despite many years of research on beta amyloid, the main component of the characteristic plaques seen in Alzheimer’s disease (AD) brains, which has been considered to be the main cause of sporadic disease, no successes in the treatment of AD that targeted beta amyloid have yet been made.

Recently there has been a growing interest in the potential role of infectious agents in the brain in the pathogenesis of AD. Strong evidence has emerged for the concept that herpes simplex virus type 1 (HSV1) is a major risk for AD when located in brain of carriers of the type 4 allele of the *APOE* gene (APOE-ε4). It has been proposed that in elderly people, the virus reactivates periodically during events such as stress and peripheral infection, thereby causing localised and limited damage in brain—both direct viral damage and viral-induced inflammation, and that accumulation of damage leads eventually to the development of AD.

This concept of a viral role was based on the discovery, some 30 years ago using PCR, that HSV1 DNA is present in a high proportion of elderly brains (4). Subsequent studies [4–14] using very diverse approaches and techniques in humans, mice and cell cultures, and currently numbering well over 400, have provided strong supportive evidence (both direct and indirect) for the concept that the virus is a major (probably causal) factor in AD.

More recently a number of other viruses have been suggested as potential causes of dementia or AD. Cytomegalovirus (CMV) seropositivity was found to correlate with dementia risk in one small study [15], although because of the very different assay sensitivities, the relative risk compared with HSV1 seropositivity was uncertain [8]. HHV6 and HHV7 were found at increased frequencies in the brains of AD patients [9] compared with those of age-matched controls but these results have been contested. Herpes zoster (shingles), which is caused by reactivation from latency of varicella zoster virus (VZV), was recently shown to increase dementia risk (though only very slightly) in a Taiwanese population [11]. Additionally, vaccinations against influenza and poliovirus were found to decrease risk of AD [12].

The relative importance of these potential viral factors in contributing to dementia remains unclear.

In this present study, we measured the correlation between seropositivity of these (and other) viruses with dementia risk in an agnostic way—that is, with no prior contextual information. We took advantage of the availability of measured antibody responses against 20 pathogens in the UK Biobank (UKB) cohort that had been selected on the basis of either being established risk factors for outcomes such as cancer, and cardiovascular or neurodegenerative diseases or of novel scientific interest (https://biobank.ctsu.ox.ac.uk/crystal/crystal/docs/infdisease.pdf). These pathogens include human herpesviruses, hepatitis B and C, polyoma, papilloma, and retroviruses. We used the seropositivity status variable (IgG) for all available viruses in UKB, with these data generated using a standardised approach. Using epidemiological data we investigated the dementia risk for each virus separately. Furthermore, we hypothised that the effect of any single virus might be small and therefore challenging to detect even in this relatively large sample. However, a combination of neurotropic viruses known to affect the central nervous system and/or found to be associated with AD might increase the effect size. To test this hypothesis we combined four such known neurotrophic viruses (HSV1, VZV, HHV6 and HHV7) and evaluated their combined effect on the risk of dementia.

In line with our hypothesis, the combined effect of the four viruses showed a statistically significant increase of dementia risk; whereas from the individually analysed viruses only HSV1 showed an association, although a weak one.

## Methods

### The study population

Information on the study population is based on data from the UK Biobank cohort (https://www.ukbiobank.ac.uk/). The UKB project is a prospective cohort study with deep genetic and phenotypic data collected on approximately 502,000 individuals from across the United Kingdom, aged between 40 and 69 at recruitment. The baseline assessment took place between 2006 and 2010 in 22 centres. At recruitment, participants provided electronic signed consent, answered questions on socio-demographic, lifestyle and health-related factors, and completed a range of physical measures. They also provided blood, urine and saliva samples [16]. Further details on participant recruitment and sample characteristics can be found in https://www.ukbiobank.ac.uk/media/gnkeyh2q/study-rationale.pdf, [17, 18].

We included 9,431 participants in our study who had available serology measurements and did not request their data to be withdrawn. This subset is a randomly selected and representative of the whole UKB cohort; however the UKB itself is not representative of the general UK population [19].

We accessed the UKB database on the 15^th^ October 2019.

### Ethical approval

The UKB obtained ethical approval l from the North West Multi-centre Research Ethics Committee (MREC) with reference number 16/NW/0274. All participants in the UK Biobank study provided fully informed consent. The project was conducted under the application number 5864.

### Serology measurement

In 2016, a pilot study was performed to determine the estimated seroprevalence of 20 infectious agents in the UKB using a validated Multiplex Serology panel. The assay was designed using the principles of an enzyme linked immunosorbent assay (ELISA) that measures levels of serum immunoglobulin G (IgG), which is a stable biological marker of lifetime exposure for a given infectious agent. The output from the Luminex reader produced quantitative data expressed in median fluorescence intensity (MFI) values per pathogen-specific antigen for each serum. The Multiplex Serology panel was designed and validated by Mentzer and colleagues, further details can be found in [20].

In the UKB seropositivity status (IgG) was available for 17 viruses.

Herpes Simplex virus-1,

Herpes Simplex virus-2,

Varicella Zoster virus,

Epstein-Barr virus,

Human Cytomegalovirus,

Human Herpesvirus-6 overall seropositivity,

Human Herpesvirus-7,

Kaposi Sarcoma-Associated Herpesvirus,

Hepatitis B virus,

Hepatitis C virus,

Human T-Lymphotropic Virus 1,

Human Immunodeficiency Virus,

Human Polyomavirus BKV,

Human Polyomavirus JCV,

Merkel Cell Polyomavirus,

Human Papillomavirus type-16 (Definition I and Definition II),

Human Papillomavirus type-18.

We used binary variables assessing seropositivity status for the 17 viruses above. Hepatitis C and human immunodeficiency virus were excluded from the analyses because of very low seroprevalence (0.31% and 0.19%, respectively, with no seropositive dementia cases in the subsample). The total number of viruses in our analysis was 15. For human papillomavirus type-16, two seropositivity definitions were available. Definition I: positive for L1 (cumulative exposure marker), definition II: positive for E6 and/or E7 (cancer marker) (https://biobank.ctsu.ox.ac.uk/crystal/crystal/docs/infdisease.pdf). Finally, for human herpesvirus-6 we used the overall seropositivity variable. These data were available for 9,431 participants. The antibodies were assayed in a blood sample taken at the initial assessment visit, at baseline between years 2006 to 2010.

### Dementia case identification

#### International Classification of Diseases (ICD) 10 and 9

The ICD 10 and 9 codes for dementia and related subgroups were obtained from the publication by Wilkinson *et al*. [21]. The ICD 10 has 212 data fields (follow-up data) and the ICD 9 has 46 data fields (follow-up data). Our analysis used data available up to 31^st^ January 2020. Information on the dates when the codes were recorded was available for each follow-up. For subjects with any of the dementia codes appearing more than once, the earliest diagnosis date was used.

Data from Primary Care linkage were available in 45% of the UKB participants at the time of this analysis. There are two versions of medical Read codes available in the UKB: version 2 (v2) and version 3 (ctV3 or v3). Both versions provide a standard vocabulary for clinicians to record patient findings and procedures, in health and social care IT systems across primary and secondary care within the National Health Service (NHS) in the UK.

### Statistical analysis

The association between seropositivity status for 15 viruses and dementia risk was assessed in a logistic regression model adjusted for age at diagnosis for dementia in the case group and age at last follow-up in the control group. Age in the regression model was fitted as a continuous variable. In this analysis we included all participants with available data on dementia status, seropositivity for 15 viruses and age (n = 9,431). The age variable was age at diagnosis for dementia cases; whilst it was defined as age at last follow up (year 2017) for non-dementia controls. The average follow-up time for the whole cohort was 8.9 years. We applied corrections for multiple testing using sharpened false discovery rate q-values [22, 23].

We then analysed the viruses that were *a priori* suggested to be neurotrophic. These were HSV1, VZV1, HHV6 and HHV7, For these viruses we developed a binary variable indicating if an individual was seropositive for all 4 of these viruses or not. We performed a logistic regression analysis using this variable together with age as an independent variables to evaluate the combined effect of the four viruses on the risk of dementia (age was defined as before). Age was fitted as a categorical variable (≤65 as reference VS >65) as presented in Table 2.

All analyses were performed using Stata SE 14 version [24].

## Results

### Demographic results

There were 9,431 participants with available antibody measures as well as dementia status data. In this subsample, the mean age of cases (age at diagnosis) was 65.4 years (SD = 8.2) and for controls was 67.0 years (SD = 8.1, age up to last follow-up). There was no statistical difference between mean age of cases and controls (*Student- t* test p-value = 0.08). There was a higher percentage of females compared to males; however the distribution of sex was not statistically significant difference (chi-square p-value = 0.732). The results are presented in Table 1.

**Table 1:** Demographic results of the sero-characterised UKB subsample.

Characteristics		Dementia controls		Dementia cases		Total	
		n	%	n	%	n	%
*Sex*	Male	4,114	44.02	39	45.88	4,153	44.04
	Female	5,232	55.98	46	54.12	5,278	55.96

### Association between seropositivity status and dementia risk

Table 2 shows the logistic regression analysis results for seropositivity status for each of the 15 viruses and dementia risk.

**Table 2:** Associations between seropositivity for 16 IgG antibody measures and dementia risk.

Virus type	Seropositivity	Dementia		Dementia				OR*	95% C.I.		P-value#
		Controls		Cases		Total					
		n	%	n	%	n	%				
*Herpes Simplex virus-1*	Negative	2,824	30.22	14	16.47	2,838	30.09	Ref			
	Positive	6,522	69.78	71	83.53	6,593	69.91	2.14	1.21	3.81	**0.010**
*Herpes Simplex virus-2*	Negative	7,832	83.8	74	87.06	7,906	83.83				
	Positive	1,514	16.2	11	12.94	1,525	16.17	0.78	0.41	1.47	0.440
*Varicella Zoster virus*	Negative	713	7.63	2	2.35	715	7.58				
	Positive	8,633	92.37	83	97.65	8,716	92.42	3.38	0.83	13.78	0.089
*Epstein-Barr virus*	Negative	493	5.27	5	5.88	498	5.28				
	Positive	8,853	94.73	80	94.12	8,933	94.72	0.9	0.36	2.24	0.823
*Human Cytomegalovirus*	Negative	3,901	41.74	37	43.53	3,938	41.76				
	Positive	5,445	58.26	48	56.47	5,493	58.24	0.89	0.57	1.37	0.587
*Human Herpesvirus-6*	Negative	866	9.27	3	3.53	869	9.21				
	Positive	8,480	90.73	82	96.47	8,562	90.79	2.79	0.88	8.85	0.082
*Human Herpesvirus-7*	Negative	501	5.36	2	2.35	503	5.33				
	Positive	8,845	94.64	83	97.65	8,928	94.67	2.4	0.59	9.8	0.222
*Kaposi Sarcoma-Associated Herpesvirus*	Negative	8,594	91.95	77	90.59	8,671	91.94				
	Positive	752	8.05	8	9.41	760	8.06	1.19	0.57	2.48	0.634
*Hepatitis B virus*	Negative	9,114	97.52	84	98.82	9,198	97.53				
	Positive	232	2.48	1	1.18	233	2.47	0.48	0.07	3.48	0.470
*Human T-Lymphotropic Virus 1*	Negative	9,200	98.44	84	98.82	9,284	98.44				
	Positive	146	1.56	1	1.18	147	1.56	0.72	0.1	5.21	0.745
*Human Polyomavirus BKV*	Negative	438	4.69	4	4.71	442	4.69				
	Positive	8,908	95.31	81	95.29	8,989	95.31	1.04	0.38	2.87	0.933
*Human Polyomavirus JCV*	Negative	3,976	42.54	34	40	4,010	42.52				
	Positive	5,370	57.46	51	60	5,421	57.48	1.12	0.73	1.74	0.599
*Merkel Cell Polyomavirus*	Negative	3,131	33.5	28	32.94	3,159	33.5				
	Positive	6,215	66.5	57	67.06	6,272	66.5	1.04	0.66	1.64	0.870
*Human Papillomavirus type-16 (Definition I)*	Negative	8,934	95.59	79	92.94	9,013	95.57				
	Positive	412	4.41	6	7.06	418	4.43	1.8	0.78	4.19	0.169
*Human Papillomavirus type-16 (Definition II)*	Negative	8,917	95.41	81	95.29	8,998	95.41				
	Positive	429	4.59	4	4.71	433	4.59	1.03	0.37	2.82	0.958
*Human Papillomavirus type-18*	Negative	9,097	97.34	83	97.65	9,180	97.34				
	Positive	249	2.66	2	2.35	251	2.66	0.93	0.23	3.79	0.914

*Adjusted for age as categorical variable (≤65 VS >65).

N.B.: (Age was also fitted as a continuous variable but this did not affect the results).

#P-value adjusted for multiple testing.

We observed a significant association between HSV1 seropositivity and dementia (OR 2.14, 95% C.I. 1.20–3.81). We further observed a marginally significant association (P-value adjusted for multiple testing) between overall HHV6 seropositivity and dementia (OR 2.78, 95% C.I. 0.88–8.84) and VZV seropositivity and dementia (OR 3.35, 95% C.I. 0.82–13.67).

Based on a combined measure of the four neurotropic viruses HSV1, VZV1, HHV6 and HHV7), 5,423 individuals were seropositive for all four, whereas 4,008 individuals were seropositive for 3, 2, 1 viruses or none at all. Logistic regression revealed that being seropositive for all 4 viruses increases the dementia risk by the OR of 2.37 (95% C.I. 1.43–3.92) (Table 3) compared the remaining group.

**Table 3:** Associations between combined 4 virus seropositivity and dementia.

	No Dementia		Dementia cases		Total		Odds ratio	95% C.I.		P-value
	n	%	n	%	N	%				
Seropositive for less than 4 viruses	3,988	42.67	20	23.53	4,008	42.50	Ref			
Seropositive for all 4 viruses	5,358	57.33	65	76.47	5,423	57.50	2.37	1.43	3.92	0.001

Using sharpened false discovery rate q-values, we find that the association between HSV1 seropositivity and dementia survives adjustment for multiple testing (q = 0.042).

## Discussion

This study investigated the relationship between viral infection and dementia. We used seropositivity status assessed for 15 viruses and found a weak statistically significant association between HSV1 infection and dementia. We also tested the hypothesis that being seropositive for four neurotropic viruses (HSV1, HHV6, HHV7 and VZV) significantly increases the risk of dementia. The literature provides evidence for these viruses causing in certain circunmstances neuropathological and immunopathological alterations in the CNS (for example HSV1: infectious encephalitis, VZV: meningitis and myelitis, HHV6: causes (rarely) encephalitis and meningitis) [25, 26].

Our results are in agreement with previous results reported in the literature. For example, a large nationwide, matched case-control study in Taiwan [10] showed that individuals with newly diagnosed HSV1 and 2 type infections had a 2.56-fold risk of developing dementia. Interestingly, a risk reduction of dementia development in patients showing severe HSV symptoms was found on treatment with anti-herpetic medications. As the study selected and treated individuals with significant clinically visible symptoms of HSV infection, it therefore differs from our study, which used serology measurements to assess HSV1 infection. The present results are broadly consistent with another Taiwan study [11] on shingles, which implicated VZV in dementia in showing that herpes zoster (shingles) conferred a risk, though very small, of dementia–HR 1.11 and that antiviral treatment of zoster sufferers greatly reduced that risk–HR 0.55.

Associations between HSV1 and HHV6, and HSV1 and CMV, though not with VZV, have been suggested previously. A PCR investigation showed that HHV6 DNA is present in elderly human brains but whether its role is additive to that of HSV1 or is independent is unknown [27]. As to CMV, Stowe and colleagues [28] proposed that the immune system of CMV-infected older adults is dysregulated, thereby leading to reactivation of HSV1. In the case of VZV, our previous study (submitted for publication) found that a history of shingles was associated with an increased risk of dementia, indicating that VZV may be involved in the development of dementia.

Interestingly, a previous examination of IgG seropositivity for HSV1, HSV2, or CMV, in home-dwelling elderly persons with cardiovascular diseases [29] showed that there was a significant association between viral infectious burden (IB) for HSV1 and CMV and cognitive impairment, assessed by the Mini-Mental Status Examination (MMSE). (There was no significant association with bacterial IB (*Chlamydia pneumoniae* and *Mycoplasma pneumoniae*)). MMSE score decreased in 150 of the 348 subjects during a 12-month follow-up. The authors suggested that the association was causal, in view of the strength of the association between viral burden and cognitive impairment, the stepwise increase in the risk of cognitive impairment with increasing viral burden, the temporal association between baseline viral burden and cognitive decline during 12 months, and the consistency of data in multiple analyses.

Letenneur et al. [30] surveyed a group of people aged 65 over a 14-year period and found, using serum anti-HSV1 IgM as a marker of recent HSV1 reactivation in the peripheral nervous system, that those who experienced HSV1 reactivation had an increased risk of developing AD compared with those who were IgM-negative. Subsequently it was shown that high HSV1-IgG antibody titres are more frequent in patients compared to age-matched controls [31]. Also, a positive correlation was reported between HSV1-specific IgG titres and the cortical volumes of brain regions mainly affected in AD, in patients with mild AD (MMSE: 18–23) [32]; it was therefore suggested that HSV1-specific humoral immunity might have a protective role in the early phase of AD. The correlation occurred only with HSV1, not with antibodies to CMV and HHV6, and there was no correlation of the latter viruses with either magnetic resonance imaging data (indicating cortical volumes) or with clinical parameters in AD patients.

Recently, Lovheim and colleagues [33] found an association between HSV carriage, as shown by the presence and level of serum IgG antibodies, and declining episodic memory function; the association was particularly strong among *APOE*-ε4 carriers. Lopatko Lindman and colleagues [34], in a large nested case-control study, found an increased risk of developing AD in the association of *APOE*-ε4 heterozygotes and anti-HSV1 IgG carriage compared with *APOE*-ε3 homozygotes, but none for carriage of either factor alone, nor for anti–HSV2 IgG, nor anti–CMV IgG. *APOE*-ε4 homozygosity increased the risk greatly, while there was no significant association with *APOE*-ε2 homozygosity. Also, a calculated genetic risk score, based on nine additional risk genes, interacted with anti–HSV1 IgG, increasing the risk of AD. Linard and colleagues [35] surveyed a prospective cohort and reported that among *APOE*-ε4 carriers–characterised by the authors as having a high frequency of HSV1 reactivation—those positive for IgM or with high IgG levels, had an increased risk of AD. *APOE*-ε4-negative subjects showed no significant association. It should be mentioned that it is uncertain whether serology data provide information about HSV1 activity and reactivation only in the peripheral nervous system, but it seems likely that they apply at least in part to HSV1 in the central nervous system also.

We chose to study the presence of IgG rather than of IgM, as the former probably provides a more reliable indicator that an individual has had exposure to a specific virus historically [20]. IgM is produced by B-cells as the first antibody to arise in response to an antigen, though after class switching, IgG predominates. The presence of IgM represents a transient response to initial viral exposure or later reactivation [30], and may be absent from most patients with a previous viral exposure. Furthermore, the IgM response can be non-specific, for example it cannot distinguish between HSV1, HSV2 or VZV [36], unlike IgG. Notably, higher levels of IgG have been proposed to function as an additional marker for viral reactivation [28, 35], presumably as the abundance of IgG-expressing B-cells after initial exposure would facilitate rapid increase in this antibody on secondary exposure.

Our study has inherent strengths and weaknesses. Its strength is the large sample size with high quality serology measurement data for a large number of different types of virus. However, we note that the dementia prevalence is lower in the UKB than in the general population. The entire UKB cohort consists of only 1.12% of all dementia cases with age of 65 and over, which is far less than the national figure prevalence of dementia—7.1%—for the total age-standardised 65+ population (based on 2013 data) [37]. The number of dementia cases in the sero-characterised subsample was 85. In general, there is evidence in the UKB cohort of healthy volunteer bias, with participants compared to the general population being less likely to be obese, to smoke, to drink alcohol on a daily basis and having fewer self-reported health conditions. Compared to nonparticipants, UKB participants tend to live in less socioeconomically deprived areas [19]. As low socioeconomic status is associated with higher prevalence of dementia [38], the higher socioeconomic status of UKB participants might be one factor behind the low number of cases in our study.

We did not take any anti-viral treatments into account, which could potentially have an effect on dementia risk if the infection occurred long before dementia diagnosis.

In conclusion, in an unbiased analysis of 15 neurotropic and non-neurotropic viruses, we found evidence that HSV1 infection, as well as the combined effect of four neurotropic viruses, (HSV1, VZV, HHV6 and HHV7) is associated with the increased risk of dementia. However, our results warrant replication in another, independent dataset.

## Abbreviations

AD: Alzheimer’s disease
CI: confidence interval
HR: hazard ratio
MFI: median fluorescence intensity
OR: odds ratio
PCR: polymerase chain reaction
SD: standard deviation
